# Solving the Power Flow Problem in Bipolar DC Asymmetric Distribution Networks Using Broyden’s Method

**DOI:** 10.3390/s23156704

**Published:** 2023-07-26

**Authors:** Oscar Danilo Montoya, Ángeles Medina-Quesada, Walter Gil-González

**Affiliations:** 1 Grupo de Compatibilidad e Interferencia Electromagnética (GCEM), Facultad de Ingeniería, Universidad Distrital Francisco José de Caldas, Bogotá 110231, Colombia; 2 Department of Electrical Engineering, University of Jaén, Campus Lagunillas s/n, Edificio A3, 23071 Jaén, Spain; 3 Department of Electrical Engineering, Universidad Tecnológica de Pereira, Pereira 660003, Colombia; wjgil@utp.edu.co

**Keywords:** Broyden’s method, power flow problem, set of nonlinear equations, linear convergence

## Abstract

This research addresses the power flow analysis in bipolar asymmetric direct current (DC) networks by applying Broyden’s numerical method. This general successive approximations method allows for a simple Newton-based recursive formula to reach the roots of multiple nonlinear equations. The main advantage of Broyden’s approach is its simple but efficient structure which can be applied to real complex nonlinear equations.The power flow problem in bipolar DC networks is still challenging, as multiple operating options must be considered, e.g., the possibility of having a solidly grounded or floating neutral wire. The main goal of this research is to contribute with a generalization of Broyden’s method for the power flow solution in bipolar DC networks, with the main advantage that, under well-defined conditions, this is a numerical method equivalent to the matricial backward/forward power flow, which is equivalent to the successive approximations power flow method. Numerical results in the 21-, 33-, and 85-bus grids while considering two connections for the neutral wire (i.e., solidly grounded at any node or floating) show the effectiveness of Broyden’s method in the power flow solution for bipolar asymmetric DC networks. All numerical simulations were carried out in the MATLAB programming environment.

## 1. Introduction

### 1.1. General Context

**Figure 1:**
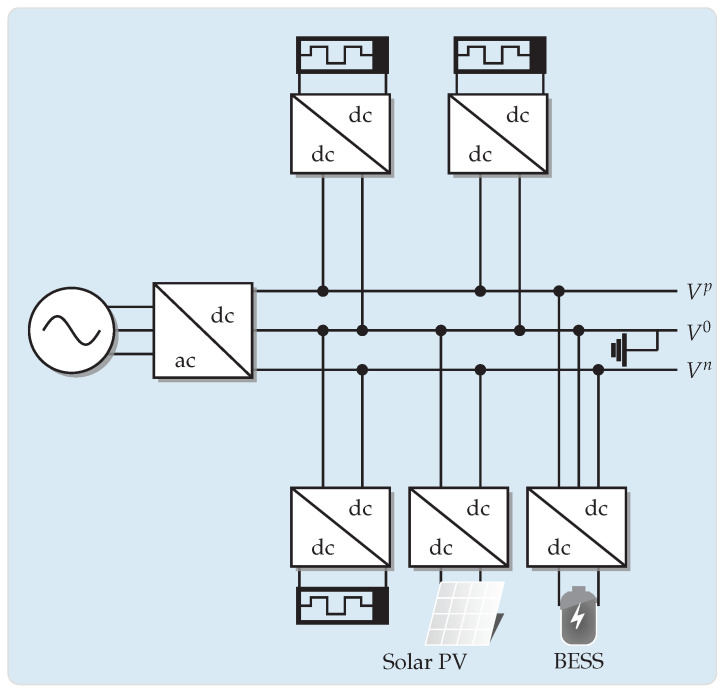
Schematic representation of a bipolar DC network.

Bipolar DC networks are emerging technologies that aid in providing electricity in medium- and low-voltage networks by using three wires associated with the positive (*p*), neutral (*o*), and negative (*n*) poles [1]. These networks can be considered to be the DC equivalent of the conventional three-phase AC distribution networks given that multiple constant power loads can be connected between their poles. These may be monopolar (connected between the positive or negative pole and the neutral wire) or bipolar (connected between the positive and negative poles) [2,3]. Figure 1 illustrates the typical configuration of a bipolar DC network with multiple constant power terminals [4].

The main characteristics shown in Figure 1 are:

- The operation of a bipolar DC network can be unbalanced due to the presence of constant monopolar and bipolar power terminals, which implies that no reductions (monopolar equivalents) must be applied [5].
- Concerning purely monopolar configurations, bipolar DC grids imply an increase of about 34% in construction investments. However, more than two times the power can be transferred by these grids, including the possible connection of exceptional loads, i.e., bipolar compositions [2].
- Depending on the operating practices of the distribution company, the neutral wire can be operated in two main ways: floating in all nodes except the substation bus or solidly grounded at any network node [6].

### 1.2. Motivation

Considering the main characteristics of bipolar DC networks, including their unbalanced operation and the possible connections of the neutral wire, power flow studies for multipolar DC networks with constant power terminals can be modeled as a set of nonlinear equations that must be solved through the application of numerical methods, as their analytical solution is impossible [7]. This research is motivated by applying Broyden’s (also known as the secant) method for finding roots in simultaneous sets of nonlinear equations [8]. The selection of this method is based on its easy formulation and implementation, as well as on its linear convergence, with the main advantage that infinite possible initial matrices $A^{t}$ (with t=0) may be selected for its numerical validation.

### 1.3. Literature Review

Several authors have focused on studying the steady-state operation of bipolar DC networks with unbalanced conditions while employing power flow analysis.

The authors of [9] proposed a power flow formulation that uses the classical nodal voltage method in order to compute the voltages and power generation of bipolar DC distribution networks while including multiple constant power terminals. Furthermore, this power flow allows considering the neutral wires in grounded or non-grounded modes. These authors only took a small bipolar DC system (three nodes) into account without analyzing the scalability of the proposed method.

The authors of study [10] used a current injection model to solve the power flow in a bipolar DC microgrid. This model was based on the Newton–Raphson power flow, which used the Jacobian matrix as index sensitivity.

The authors of [1] described a generic power flow algorithm for bipolar DC grids that employs the Newton–Raphson method. This method classifies the grid nodes into six types based on their grounding scheme and voltage control mode. This classification identifies the unknown pole voltages which are used as mismatch equations to solve the power flow problem. One of the limitations of this generic power flow algorithm is that it is not possible to establish the non-convergence condition.

The authors of [11] analyzed a power flow method under different operation modes and control strategies in bipolar high-voltage DC (HVDC) grids integrated with voltage source converters (VSCs). The power flow method was based on layers and the admittance matrix of the bipolar grid.

The authors of study [7] proposed a successive approximations method to solve power flow in bipolar DC distribution networks. This method allows considering a grounded or non-grounded neutral wire.

The authors of study [3] demonstrated the exact conditions and necessities for the convergence and uniqueness of the power analysis solution in bipolar DC grids. This study also considered the over-current protection scheme of power electronic converters.

The authors of work [6] tackled the power flow problem in bipolar DC grids with multiple constant power loads by employing a triangular-based power flow formulation. This formulation is suitable for radial setups and considers whether the neutral wire is grounded or not at all system nodes.

The authors of study [7] used a successive approximations technique based on the classical backward/forward method to solve the power flow analysis in bipolar DC grids, including the possibility of grounding or not grounding the neutral wire. Furthermore, the convergence of the proposed approximation was demonstrated using the Banach fixed-point theorem.

The authors of [12] presented a formulation to solve the power flow problem in bipolar DC networks, which used the Taylor expansion in the bus injection equations, generating a hyperbolic formulation that can be implemented in both meshed and radial networks.

Previous studies have shown promising results, but some of them struggle to achieve convergence in their performance. Additionally, some of these studies lack a comprehensive framework to address these limitations. This paper presents a solution to the power flow problem in bipolar DC asymmetric distribution networks using Broyden’s method. This method is a quasi-Newton method that numerically solves systems of nonlinear equations through iterative processes. In addition, it offers several advantages over other approaches [13,14]. For example, this method provides greater versatility, making it applicable to a broader range of problems without requiring additional modifications. Additionally, Broyden’s method has demonstrated good and rapid convergence. Moreover, it is robust, as it can be implemented in cases where other techniques, such as Newton’s method, may encounter difficulties due to ill-conditioned Jacobian matrices.

### 1.4. Contributions and Scope

Considering the state of the art in numerical methods applied to the solution of the power flow problem in bipolar DC networks with asymmetric loading, the main contributions of this research are the following:

- The application of Broyden’s method to the power flow problem in bipolar DC grids with the aim to solve the set of Equations $\nabla f\left(x\right)=0$ by using different $A^{t}$ matrices.
- The evaluation of the convergence properties of Broyden’s method using random initialization matrices $A^{t}$ with t=0, as well as an analysis of its equivalence with the successive approximations method when $A^{0}$ is selected as part of the conductance matrix of the system.
- A demonstration of the equivalence between the successive approximations power flow (SAPF) and Broyden’s method, which proves that the former is a particular case of the latter in the studied problem.

Regarding the scope of our contribution, the following facts are considered during the numerical validations carried out in the 21-, 33-, and 85-bus grids: (i) two possible connections for the neutral wire are tested: the first case corresponds to the floating connection, i.e., the neutral wire is only solidly grounded at the substation terminals, and the second case assumes that all the nodes are solidly grounded; (ii) the load consumption values are assumed to be perfectly known, i.e., no uncertainties are considered in the values of the constant power terminals; and (iii) the voltage output at the substation bus for all the poles is fixed as an input parameter.

### 1.5. Document Structure

The remainder of this document is structured as follows. Section 2 presents the general formulation of the power flow problem for bipolar DC networks with asymmetric loading. Section 3 describes the general implementation of Broyden’s method for a general set of nonlinear equations. Section 4 presents the application of Broyden’s method to the power flow problem. Section 5 shows the main characteristics of the test feeders, which correspond to the 21-, 33-, and 85-bus grids. Section 6 presents the numerical validations of the proposed power flow problem, including the voltage profile performance and the power losses for each test feeder, as well as some numerical comparisons against the literature reports. Finally, Section 7 lists the main concluding remarks derived from this work and some possible future studies.

## 2. Power Flow Problem in Bipolar DC Networks

The power flow solution for bipolar DC networks with asymmetric loading is a challenge due to the presence of multiple nonlinear equality constraints associated with each pole as a function of the connection of multiple constant power loads (monopolar or bipolar) [15]. This set of constraints requires implementing numerical methods in order to obtain a solution with an acceptable convergence error [1]. The power flow problem in a bipolar unbalanced network can be represented with seven equations (three linear and four nonlinear) per node and pole. These equations are presented below.

(1) $$\begin{array}{c} I_{g,k}^{p}-I_{d,k}^{p}-I_{d,k}^{p-n}=\sum_{r\in P}\sum_{j\in N}G_{jk}^{pr}V_{k}^{r},\;\left\{\forall k\in N\right\}, \end{array}$$

(2) $$\begin{array}{c} I_{g,k}^{o}-I_{d,k}^{o}-I_{d,k}^{\mathrm{ground}}=\sum_{r\in P}\sum_{j\in N}G_{jk}^{or}V_{k}^{r},\;\left\{\forall k\in N\right\}, \end{array}$$

(3) $$\begin{array}{c} I_{g,k}^{n}-I_{d,k}^{n}+I_{d,k}^{p-n}=\sum_{r\in P}\sum_{j\in N}G_{jk}^{nr}V_{k}^{r},\;\left\{\forall k\in N\right\}, \end{array}$$

(4) $$\begin{array}{c} I_{d,k}^{p}=\frac{P_{d,k}^{p}}{V_{k}^{p}-V_{k}^{o}},\;\left\{\forall k\in N\right\}, \end{array}$$

(5) $$\begin{array}{c} I_{d,k}^{n}=\frac{P_{d,k}^{n}}{V_{k}^{n}-V_{k}^{o}},\;\left\{\forall k\in N\right\}, \end{array}$$

(6) $$\begin{array}{c} I_{d,k}^{o}=\frac{P_{d,k}^{p}}{V_{k}^{o}-V_{k}^{p}}+\frac{P_{d,k}^{n}}{V_{k}^{o}-V_{k}^{n}},\;\left\{\forall k\in N\right\}, \end{array}$$

(7) $$\begin{array}{c} I_{d,k}^{p-n}=\frac{P_{d,k}^{p-n}}{V_{k}^{p}-V_{k}^{n}},\;\left\{\forall k\in N\right\}, \end{array}$$

where $I_{g,k}^{p}$, $I_{g,k}^{o}$, and $I_{g,k}^{n}$ represent the current injection in the slack source per pole (i.e., positive, neutral, and negative -pon-); $I_{d,k}^{p}$, $I_{d,k}^{o}$, and $I_{d,k}^{n}$ are the current demanded by the monopolar constant power loads, i.e., $P_{d,k}^{p}$ and $P_{d,k}^{n}$ (note that the monopolar loads refer to those connected between the positive or negative pole and the neutral pole); $I_{d,k}^{p-n}$ corresponds to the current absorbed by the constant power load $P_{d,k}^{p-n}$ connected between the positive and negative poles, i.e., a bipolar consumption; $I_{d,k}^{\mathrm{ground}}$ means the current drained from the neutral wire to the physical ground at each node; $V_{k}^{r}$ represents the voltage magnitude at node *k* in pole *r*; $G_{jk}^{pr}$, $G_{jk}^{or}$, and $G_{jk}^{nr}$ correspond to the conductance values (obtained from the conductance matrix) that relate nodes *j* and *k* and poles pon with pole *r*, respectively. Note that P and N denote the sets that contain all the poles and nodes of the bipolar DC grid under analysis.

If Equations (4)–(7) are substituted into (1)–(3), the studied problem is reduced to a set of three nonlinear equality constraints.

(8) $$\begin{array}{c} I_{g,k}^{p}-\frac{P_{d,k}^{p}}{V_{k}^{p}-V_{k}^{o}}-\frac{P_{d,k}^{p-n}}{V_{k}^{p}-V_{k}^{n}}=\sum_{r\in P}\sum_{j\in N}G_{jk}^{pr}V_{k}^{r},\;\left\{\forall k\in N\right\}, \end{array}$$

(9) $$\begin{array}{c} I_{g,k}^{o}-\frac{P_{d,k}^{p}}{V_{k}^{o}-V_{k}^{p}}+\frac{P_{d,k}^{n}}{V_{k}^{o}-V_{k}^{n}}-I_{d,k}^{\mathrm{ground}}=\sum_{r\in P}\sum_{j\in N}G_{jk}^{or}V_{k}^{r},\;\left\{\forall k\in N\right\}, \end{array}$$

(10) $$\begin{array}{c} I_{g,k}^{n}-\frac{P_{d,k}^{n}}{V_{k}^{n}-V_{k}^{o}}+\frac{P_{d,k}^{p-n}}{V_{k}^{p}-V_{k}^{n}}=\sum_{r\in P}\sum_{j\in N}G_{jk}^{nr}V_{k}^{r},\;\left\{\forall k\in N\right\}. \end{array}$$

**Remark 1.**  *The set of nonlinear Equations *(8)–(10)* can be compacted using a matricial representation with the help of the conductance matrix and its components $G_{gg}$, $G_{gd}$, $G_{dg}$, and $G_{dd}$, as defined in *(11)* [7].*

(11) $$\left[\begin{array}{c} I_{g}\\ -I_{d} \end{array}\right]=\left[\begin{array}{cc} G_{gg} & G_{gd}\\ G_{dg} & G_{dd}\; \end{array}\right]\left[\begin{array}{c} V_{g}\\ V_{d} \end{array}\right],$$

*where, considering that the cardinalities of the sets P and N are 3 and n, $I_{g}\in R^{3\times 1}$ and $V_{g}\in R^{3\times 1}$ are the vectors that contain the current injection in the slack source (unknown variables) and the voltage output at the terminals of the slack source, i.e., perfectly known voltages. $I_{d}\in R^{3(n-1)\times 1}$ is the vector containing the current demanded at each node, which is ordered by node and pole (unknown variables); and $V_{d}\in R^{3(n-1)\times 1}$ is the vector of voltage profiles at each demand node, also ordered by node and pole (unknown variables).*

From the two equations in (11), it can be noted that:

- The first row of (11) shows that the solution of the current injections at the substation will be only known when all the demanded voltage profiles are determined. In addition, this is a linear equation that does not require any iterative process for its solution.
- The second row of (11) is a nonlinear set of equations, as the demanded currents $I_{d}$ are hyperbolic functions of the demanded voltages, which implies that its solution requires efficient numerical methods [1].

To determine the set of demanded currents by comparing the second row of (11) against Equations (8)–(10), note that

$$\begin{array}{c} I_{d}=I_{d}^{B}+I_{d}^{M}, \end{array}$$

where $I_{d}^{B}$ and $I_{d}^{M}$ are vectors associated with the currents demanded by the bipolar and monopolar loads, respectively, which are calculated per node, as follows:

(12) $$\begin{array}{c} I_{d,k}^{B}={\mathrm{diag}}^{-1}\left(BV_{d,k}\right)P_{d,k}^{B},\;\left\{\forall k\in N\right\}, \end{array}$$

(13) $$I_{d,k}^{M}={\mathrm{diag}}^{-1}\left(M_{1}V_{d,k}\right)P_{d,k}^{M_{1}}+{\mathrm{diag}}^{-1}\left(M_{2}V_{d,k}\right)P_{d,k}^{M_{2}},\;\left\{\forall k\in N\right\},$$

where

$$\begin{array}{c} B=\left[\begin{array}{ccc} 1 & 0 & -1\\ 0 & 1 & 0\\ -1 & 0 & 1 \end{array}\right],\;M_{1}=\left[\begin{array}{ccc} 1 & -1 & 0\\ -1 & 1 & 0\\ 0 & -1 & 1 \end{array}\right],\;M_{2}=\left[\begin{array}{ccc} 0 & 0 & 0\\ 0 & 1 & -1\\ 0 & 0 & 0 \end{array}\right],\\ P_{d,k}^{B}=\left[\begin{array}{c} P_{d,k}^{p-n}\\ 0\\ P_{d,k}^{p-n} \end{array}\right],\;P_{d,k}^{M_{1}}=\left[\begin{array}{c} P_{d,k}^{p}\\ P_{d,k}^{p}\\ P_{d,k}^{n} \end{array}\right],\;P_{d,k}^{M_{2}}=\left[\begin{array}{c} 0\\ P_{d,k}^{n}\\ 0 \end{array}\right],\;V_{d,k}=\left[\begin{array}{c} V_{d,k}^{p}\\ V_{d,k}^{o}\\ V_{d,k}^{n} \end{array}\right]. \end{array}$$

**Remark 2.**  *The solution to the power flow problem in bipolar DC grids with asymmetric loading corresponds to the solution of the following recursive formula:*

(14) $$\begin{array}{c} G_{dg}V_{g}+G_{dd}V_{d}=-I_{d}^{B}-I_{d}^{M} \end{array}$$

*using a numerical method based on the dependence of the vectors $I_{d}^{B}$ and $I_{d}^{M}$ on the demanded voltages $V_{d}$.*

## 3. The Secant or Broyden’s Method

The secant or Broyden’s method is a numerical approach developed for solving sets of nonlinear equations with $\nabla f\left(x\right)=0$. This is performed without resorting to the implementation of Newton-based approaches [8,16]. The general successive approximations formula for the secant method is defined in (6).

(15) $$\begin{array}{c} x^{t+1}=x^{t}-{\left[A^{t}\right]}^{-1}\nabla f\left(x^{t}\right), \end{array}$$

where *t* is the iteration counter and $A^{t}$ corresponds to the Jacobian matrix, which is, however, approximated via secant hyperplanes [17].

Consider the following linear relation:

(16) $$\begin{array}{c} A^{t}\left(x^{t}-x^{t-1}\right)=\nabla f\left(x^{t}\right)-\nabla f\left(x^{t-1}\right), \end{array}$$

which is simplified as (17) by defining $s^{t}=\left(x^{t}-x^{t-1}\right)$ and $y^{t}=\nabla f\left(x^{t}\right)-\nabla f\left(x^{t-1}\right)$.

(17) $$\begin{array}{c} A^{t}s^{t}=y^{t}. \end{array}$$

Now, the following two hyperplanes are defined:

(18) $$\begin{array}{c} l_{t-1}\left(x\right)=\nabla f\left(x^{t-1}\right)+\left[A^{t-1}\right]\left(x-x^{t-1}\right), \end{array}$$

(19) $$\begin{array}{c} l_{t}\left(x\right)=\nabla f\left(x^{t}\right)+\left[A^{t}\right]\left(x-x^{t}\right). \end{array}$$

The main idea of the secant method is that, at the solution point, hyperplanes (18) and (19) must be equal, i.e.,

(20) $$\begin{array}{c} \nabla f\left(x^{t-1}\right)+\left[A^{t-1}\right]\left(x-x^{t-1}\right)=\nabla f\left(x^{t}\right)+\left[A^{t}\right]\left(x-x^{t}\right). \end{array}$$

Note that, in (20), if $x=x^{t}$ is defined while considering the definition in (17) and making some algebraic manipulations, the following result is reached:

(21) $$\begin{array}{c} \left(\left[A^{t}\right]-\left[A^{t-1}\right]\right)s^{t}=y^{t}-\left[A^{t-1}\right]s^{t}. \end{array}$$

By simplifying (21), the general updating formula for $A^{t}$ can be determined as presented in (22).

(22) $$\begin{array}{c} A^{t}=A^{t-1}-\frac{\left(y^{t}-\left[A^{t-1}\right]s^{t}\right){\left(s^{t}\right)}^{⊤}}{{\left(s^{t}\right)}^{⊤}s^{t}}. \end{array}$$

Note that, for the general iteration t+1, the way to update $A^{t+1}$ is obtained from (22), as defined in (23).

(23) $$\begin{array}{c} A^{t+1}=A^{t}+\frac{\left(y^{t+1}-\left[A^{t}\right]s^{t+1}\right){\left(s^{t+1}\right)}^{⊤}}{{\left(s^{t+1}\right)}^{⊤}s^{t+1}}. \end{array}$$

The general algorithm for solving the set of equations $\nabla f\left(x\right)=0$ is presented in Algorithm 1 [18].
**Algorithm 1:** General implementation of Broyden’s method for solving sets of nonlinear equations
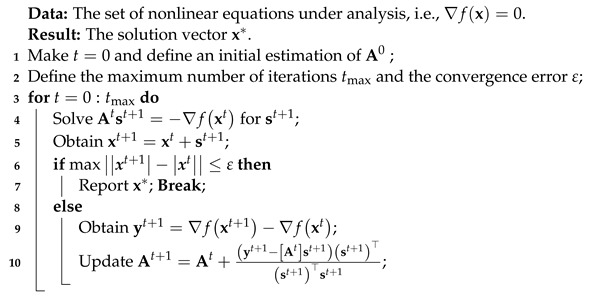


## 4. Application of Broyden’s Method to the Power Flow Problem

This section presents the general application of Broyden’s method to the problem regarding the power flow of bipolar DC networks with asymmetric loading, which is based on Algorithm 1. As presented in Section 2, this work deals with a complex nonlinear problem, where the main idea is to efficiently obtain its roots via numerical methods [1]. For the application of Broyden’s method, as described in Section 3, using the compact representation in Equation (14) is recommended. Note that this Equation is necessary as the equivalent gradient function $\nabla f\left(V_{d}^{t}\right)$ is defined from it, as follows [18]:

(24) $$\begin{array}{c} \nabla f\left(V_{d}^{t}\right)=G_{dg}V_{g}+G_{dd}V_{d}^{t}+I_{d}^{t,B}+I_{d}^{t,M}=0, \end{array}$$

where the components of the demanded currents in $I_{d}^{t,B}$ and $I_{d}^{t,M}$ are defined from (12) to (13).

Considering the structure of Algorithm 1, Algorithm 2 presents the application of Broyden’s method to solve the power flow problem in asymmetric bipolar DC distribution grids.

**Remark 3.**  *The main characteristic of Algorithm 2 is the selection of the initial value of matrix $A^{0}$, as it has multiple alternatives. Depending on this selection, the number of iterations required to reach the desired convergence varies.*

**Algorithm 2:** Application of Broyden’s method for solving the power flow problem in bipolar DC networks

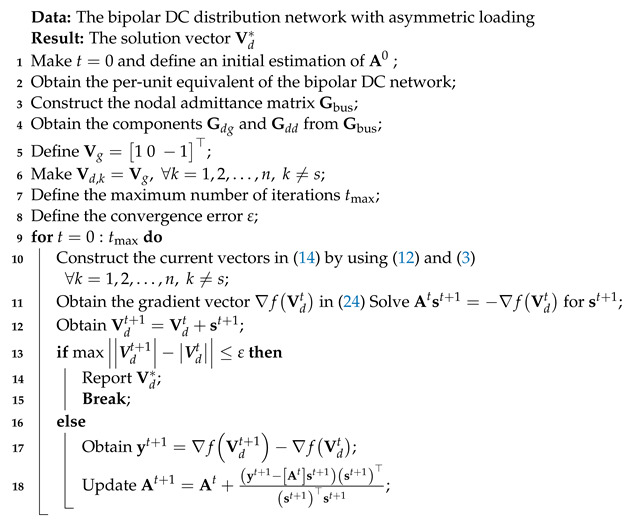



## 5. Test Feeders

To validate the effectiveness of Broyden’s method in addressing the power flow problem in bipolar asymmetric distribution grids, three test feeders composed of 21, 33, and 85 nodes with unbalanced structures were considered.

### 5.1. Bipolar DC 21-Bus Network

**Figure 2:**
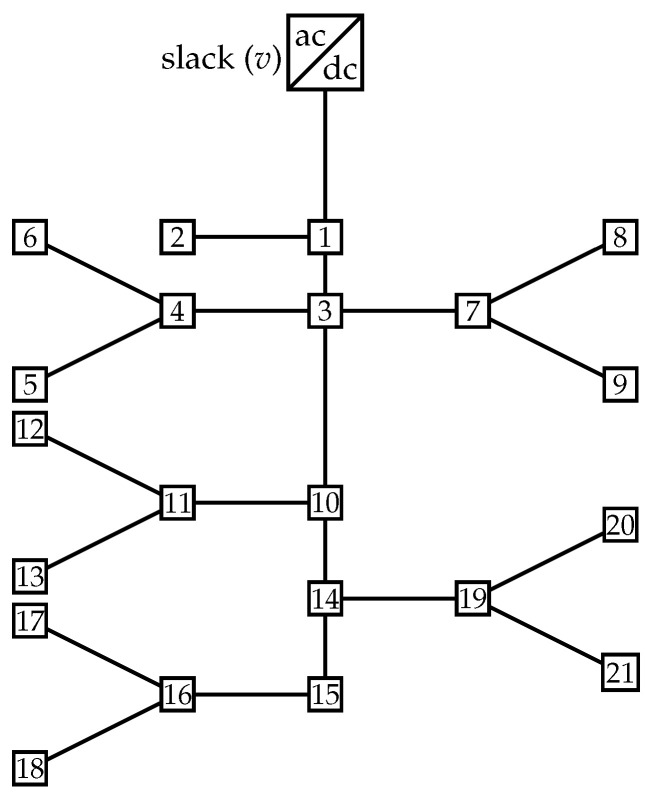
Schematic of nodal connections in the bipolar DC 21-bus network.

The 21-bus grid corresponds to a radial distribution network composed of 21 buses and 20 lines, as depicted in Figure 2 [6]. The main characteristics of this feeder are the following: (i) the substation bus is located at bus 1, and the voltage outputs in the positive and negative poles are ±1 kV with respect to the neutral point, which is solidly grounded; and (ii) the total monopolar consumption is 554 kW in the positive pole and 445 kW in the negative pole, while the bipolar power consumption is 405 kW.

The information regarding branches and monopolar and bipolar power consumptions is presented in Table 1.

### 5.2. Bipolar DC 33-Bus System

**Figure 3:**
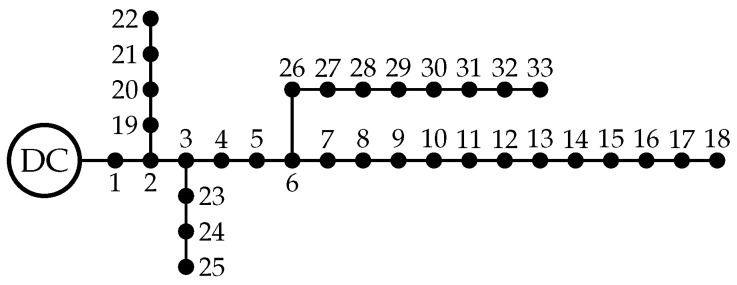
Schematic of the nodal connections in the bipolar DC 33-bus network.

This bipolar DC network is a modification of the original single-phase distribution network originally proposed by the authors of [19]. The schematic single-line representation of this system is presented in Figure 3. The main characteristics of this distribution network are the following: (i) the substation bus is located at bus 1, and the voltage outputs in the positive and negative poles are ±12.66 kV with respect to the neutral point, which is solidly grounded; and (ii) the total monopolar consumption is 2615 kW in the positive pole and 2185 kW in the negative pole, while the bipolar power consumption is 2350 kW.

The information regarding branches and monopolar and bipolar power consumptions is presented in Table 2 [20].

### 5.3. Bipolar DC 85-Bus Network

**Figure 4:**
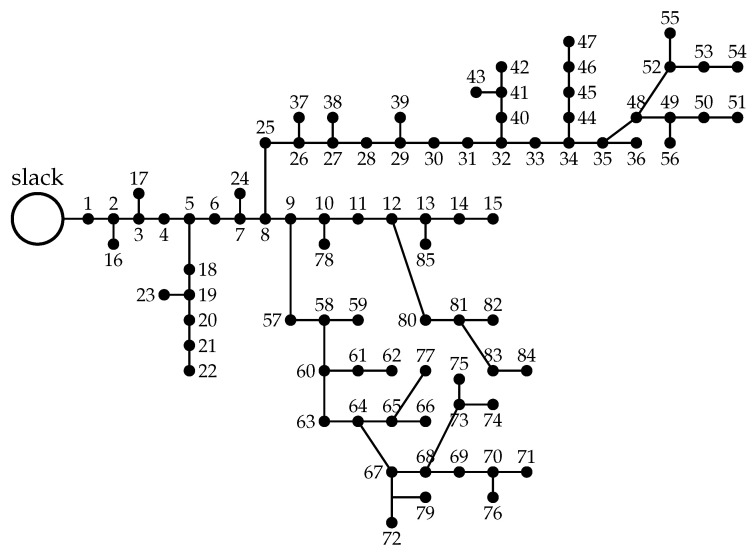
Schematic of the nodal connections in the bipolar DC 85-bus network.

The bipolar DC 85-network is a radial distribution network composed of 85 nodes and 84 branches, as depicted in Figure 4. The main characteristics of this distribution network are the following: (i) the substation bus is located at bus 1, and the voltage outputs in the positive and negative poles are ±11 kV with respect to the neutral point, which is solidly grounded; and (ii) the total monopolar consumption is 1745.48 kW in the positive pole and 2682.19 kW in the negative pole, while the bipolar power consumption is 2258.58 kW.

The information regarding branches and monopolar and bipolar power consumptions is presented in Table 3 [21].

## 6. Numerical Validations

To validate Broyden’s method, all the bipolar DC networks were simulated in the MATLAB programming environment via the researchers’ own scripts. For this implementation, version 2021b was used on a PC with an AMD Ryzen 7 3700 2.3-GHz processor and 16.0 GB RAM, running on a 64-bit version of Microsoft Windows 10 Single language.

### 6.1. Number of Iterations and Convergence Behavior

**Figure 5:**
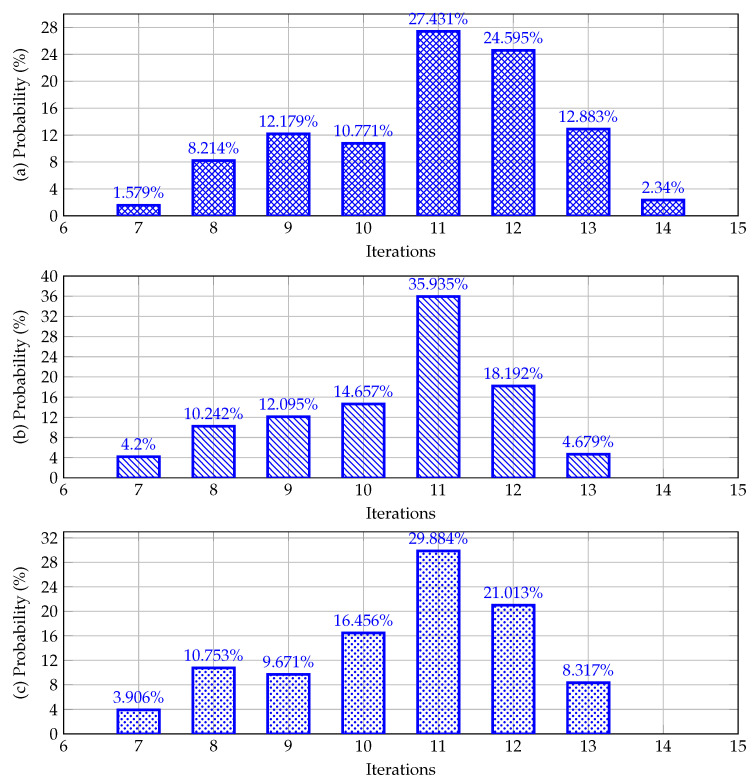
Behavior of the iterations as a function of the initial matrix $A^{0}$: (**a**) bipolar DC 21-bus network, (**b**) bipolar DC 33-bus network, and (**c**) bipolar DC 85-bus network.

In this section, the $A^{0}$ matrix is generated using the conductance matrix $G_{dd}$ with the following rule: $A^{0}=\gamma G_{dd}$, where $\gamma =\alpha +\mathrm{rand}(1)\left(\beta -\alpha \right)$. rand(1) is a random number generated using a uniform distribution, and the parameters α and β are set as 0.5 and 2.0, respectively. In addition, to test the variations in the number of iterations required to reach the power flow solution while considering a convergence error of about $\varepsilon =1\times 10^{-10}$, for these simulations, it is assumed that the neutral wire is floating in all nodes except in the substation bus. The number of iterations required for solving the power flow problem with Broyden’s method in each one of the test feeders is depicted in Figure 5.

The main characteristics of the behavior shown in Figure 5 allow stating that:

- The number of iterations after 100,000 repetitions exhibits a Gaussian distribution, with a mean value of about 11 iterations for all the test feeders. This is due to the fact that the Gaussian distribution carried most of the γ-factor around its center, i.e., $\frac{\alpha +\beta }{2}=1.25$, which is the region where 11 iterations are the most probable result.
- Depending on the γ-factor, for all the test feeders, a minimum number of iterations of about seven was observed. The average values of the γ-factor were 0.9750, 0.9659, and 0.9783, for the bipolar DC 21-, 33-, and 85-bus networks, respectively. These values imply that in order to reach a better numerical performance with Broyden’s method, the γ-factor must be tuned for each test feeder.
- The maximum number of iterations for the 21-bus grid was about 14, whereas for the 33- and 85-bus grids it was 13. For these results, the γ-factor is when this parameter is located near the α of β values, i.e., near the extreme values used in simulations.

**Figure 6:**
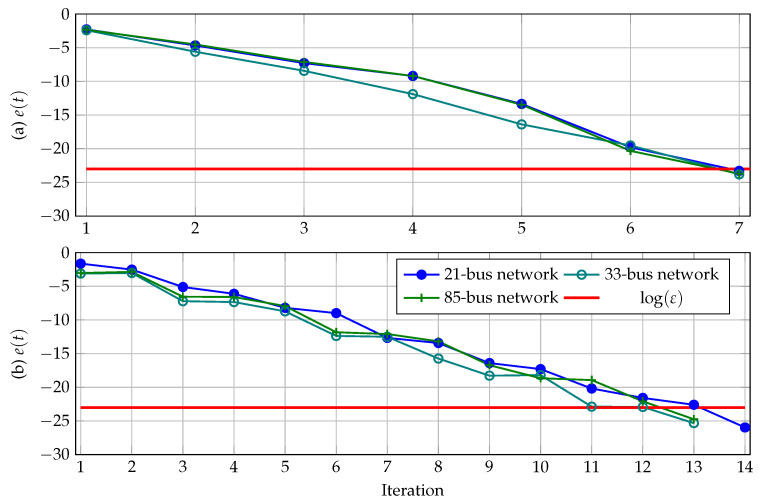
Convergence behavior of Broyden’s method for all the tested feeders: (**a**) when the γ-factor is selected to ensure the minimum number of iterations, and (**b**) when the γ-factor is selected to ensure the maximum number of iterations.

To determine the convergence properties of Broyden’s method in all the test feeders under analysis, a logarithmic error graph is presented in Figure 6. Equation (25) presents the function that defines the error’s evolution.

(25) $$\begin{array}{c} e\left(t\right)=\log\left(\left|\left|V_{d}^{t+1}\right|-\left|V_{d}^{t}\right|\right|\right). \end{array}$$

The convergence behavior exhibited by Broyden’s method in Figure 6 for all the tested feeders when the γ-factor was selected to ensure the minimum or the maximum number of iterations within the range assigned for α and β shows that, in general, this algorithm has a linear tendency to solve the power flow problem, with some oscillations. However, these oscillations are caused by the updating of the $A^{t}$ matrix, which is carried out by using the information of the previous iteration. Still, this does not compromise the stability of this method.

**Remark 4.**  *The γ-factor is a key parameter in the implementation of Broyden’s method. However, as observed for all the test feeders, the recommended value to ensure the minimum number of iterations must be contained in the 0.90–1.10 interval.*

### 6.2. Comparative Analysis

To demonstrate the effectiveness of the proposed secant method to deal with the power flow problem in bipolar DC asymmetric distribution networks, the power flow was solved while considering the two connection modes of the neutral wire (i.e., solidly grounded or floating) in the tested feeders. A comparison was made with the hyperbolic approximations power flow (HAPF) approach reported in [6], the successive approximations power flow (SAPF) method proposed by [7], and the triangular-based power flow (TBPF) approach reported in [12]. Table 4 lists the results for all the test feeders, including the two connections applicable to the neutral wire. For the sake of simplicity, the gamma factor was selected to ensure minimal iterations in Broyden’s power flow method.

The numerical results in Table 4 show that:

- In all simulation cases, Broyden’s method reaches the same numerical solution regarding power losses in the tested feeders for both neutral wire operating conditions. Nevertheless, when the neutral wire is floating, this approach takes 7 iterations; otherwise, 11 iterations are needed. These values were independent of the number of nodes of the test feeder under analysis.
- Contrary to the behavior of the comparison methods, the number of iterations increases when the neutral wire is considered to be solidly grounded. However, this behavior can be attributed to the fact that Broyden’s recursive formula is general and does not depend on the nonlinear set of equations under analysis, which implies that, under some particular conditions (i.e., the connection of the neutral wire), its evolution differs from that of specialized methods for power flow studies. On the other hand, the increase in the number of iterations is not directly related to that of the required processing times.
- The main characteristic of the simulations in the three test feeders is that (as expected) when the neutral wire is solidly grounded, the power losses are lower than in the floating operation scenario. These differences are 4.1536, 10.0629, and 37.2778 kW, for the 21-, 33-, and 85-bus networks, respectively.

On the other hand, the behavior of the voltage profiles in the tested feeders shows voltage regulations of 11.1740%, 9.4264%, and 7.9629% for the neutral floating case in the 21-, 33-, and 85-bus networks, as well as 10.9898%, 9.3176%, and 8.1086% when the neutral wire is solidly grounded. As expected, voltage regulation improves when the neutral wire is solidly grounded, which is a direct consequence of the reduction in the number of power losses evidenced in Table 4.

### 6.3. Demonstration of the Equivalence between the SAPF and Broyden’s Method

The main characteristic of Broyden’s method is that it can reduce the number of calculations (iterations) required to solve the power flow problem in bipolar DC networks under floating operating conditions. However, the time taken by Broyden’s method is superior to that of the SAPF method, as the former needs to update the $A^{t}$ matrix. Although it is feasible under some particular conditions, Broyden’s method is equivalent to the SAPF approach. This equivalence is demonstrated below.

**Lemma 1.**  *The SAPF method is an iterative procedure to solve the power flow problem in electrical networks which can be obtained from Equation *(14)*, as demonstrated in [7], which takes the following form:*

(26) $$\begin{array}{c} V_{d}^{t+1}=-G_{dd}^{-1}\left(G_{dg}V_{g}+I_{d}^{t,B}+I_{d}^{t,M}\right), \end{array}$$

*which converges if the $G_{dd}$ is a diagonally dominant matrix and the bipolar DC network operates far from the voltage collapse point [22].*

To demonstrate that Broyden’s method is equivalent to the SAPF approach, let us consider the following assumptions:

**Assumption A1.**  *The γ-factor is selected as the unity, which implies that $A^{0}=G_{dd}$.*

**Assumption A2.**  *No updating is implemented for the $A^{t+1}$ matrix, i.e., $A^{t+1}=A^{t}=A^{0}$.*

**Proof.**  Considering Assumptions 1 and 2, the general evolution rule of Broyden’s method in Equation (15) takes the following form:

(27) $$\begin{array}{c} x^{t+1}=x^{t}-{\left[A^{0}\right]}^{-1}\nabla f\left(x^{t}\right). \end{array}$$

Now, considering that, for the bipolar DC power flow problem $f\left(x^{t}\right)=f\left(V_{d}^{t}\right)$, and given the definition in (24), Equation (27) takes the following form:

(28) $$\begin{array}{cc} V_{d}^{t+1} & =V_{d}^{t}-{\left[A^{0}\right]}^{-1}\nabla f\left(V_{d}^{t}\right),\\ \; & =V_{d}^{t}-{\left[A^{0}\right]}^{-1}\left(G_{dg}V_{g}+G_{dd}V_{d}^{t}+I_{d}^{t,B}+I_{d}^{t,M}\right). \end{array}$$

If the definition of $A^{0}$ is taken into account, Equation (28) can be simplified as follows:

(29) $$\begin{array}{cc} V_{d}^{t+1} & =V_{d}^{t}-G_{dd}^{-1}\left(G_{dg}V_{g}+G_{dd}V_{d}^{t}+I_{d}^{t,B}+I_{d}^{t,M}\right),\\ \; & =V_{d}^{t}-G_{dd}^{-1}\left(G_{dg}V_{g}+I_{d}^{t,B}+I_{d}^{t,M}\right)-V_{d}^{t},\\ \; & =-G_{dd}^{-1}\left(G_{dg}V_{g}+I_{d}^{t,B}+I_{d}^{t,M}\right), \end{array}$$

which allows noting that Equation (29) is identical to the SAPF formula in Equation (26), thus completing the proof. □

**Remark 5.**  *It is important to highlight that this proof considers that the SAPF method is a particular case of Broyden’s method for analyzing bipolar DC asymmetric networks.*

## 7. Conclusions

This research applied Broyden’s method to solve the power flow problem in bipolar DC asymmetric distribution networks while considering two possible connections for the neutral pole, i.e., solidly grounded or floating case. Numerical results demonstrated that:

- The number of iterations required by the proposed numerical method to solve the studied problem highly depends on the γ factor selection. This parameter must be tuned for each test feeder and neutral wire connection, with its recommended values being between 0.90 and 1.10. Note that the γ-coefficient can be considered to be the equivalent value of the α-coefficient in the classical Gauss–Seidel method implemented for AC power systems.
- The convergence behavior, i.e., the evolution of $e\left(t\right)$, exhibits a linear behavior as a function of the selected γ-factor, with slight oscillations attributed to the updating rule applicable to Broyden’s method, which adds the effect of the difference between two consecutive nonlinear function evaluations to the calculation of $A^{t}$. In addition, after 100 thousand consecutive evaluations of Broyden’s method for different values of the γ-factor, it was observed that the highest probability in the expected number of iterations was 11, regardless of the test feeder, with a minimum value 7 for all test systems, as well as a maximum of 14 for the 21-bus grid and 13 for the remaining grids.
- Numerical comparisons with the literature reports showed that the processing times in the solution of the power flow increase with the number of nodes of the network, as expected. However, regarding the number of iterations, the opposite behavior was evidenced in the methods used for comparison (SAPF, TBPF, and HAPF): in the case of the floating neutral wire, the number of iterations was lower for our method, and, when the neutral wire was solidly grounded, the number of iterations increased. This, however, was inversely related to the average processing times.
- A demonstration of the equivalence between the SAPF approach and Broyden’s method under Assumptions 1 and 2 confirmed the generality of the proposed technique in dealing with power flow problems in bipolar DC networks with asymmetric loads, with the main advantage that the number of iterations or processing times can be prioritized as a function of the γ-factor and the selection and adapting of the $A^{t+1}$ matrix.

The main advantage of Broyden’s method for addressing the power flow problem in bipolar DC networks with asymmetric loading is that it can apply to radial and meshed networks as an equivalent to the SAPF and the HAPF approaches given that its formulation is based on a conductance matrix that is constructed without considering the tree configuration of the network under analysis. However, numerical tests were only conducted in three radial distribution networks since these configurations correspond to the worst possible case regarding power losses and voltage regulation indicators.

In regard to future works, the following studies can be conducted: (i) extending the proposed power flow method to three-phase asymmetric distribution networks; (ii) including, in Broyden’s formulation, the possibility of having multiple voltage-controlled nodes and distributed energy resources; and (iii) demonstrating the equivalence between the SAPF method and the proposed approach.

## Figures and Tables

**Table 1:** Parametric information regarding branches and constant power loads in the bipolar DC 21-bus network (all powers in kW).

Node *j*	Node *k*	$R_{\mathrm{jk}}$ (Ω)	$P_{d,k}^{p}$	$P_{d,k}^{n}$	$P_{d,k}^{p-n}$
1	2	0.053	70	100	0
1	3	0.054	0	0	0
3	4	0.054	36	40	120
4	5	0.063	4	0	0
4	6	0.051	36	0	0
3	7	0.037	0	0	0
7	8	0.079	32	50	0
7	9	0.072	80	0	100
3	10	0.053	0	10	0
10	11	0.038	45	30	0
11	12	0.079	68	70	0
11	13	0.078	10	0	75
10	14	0.083	0	0	0
14	15	0.065	22	30	0
15	16	0.064	23	10	0
16	17	0.074	43	0	60
16	18	0.081	34	60	0
14	19	0.078	9	15	0
19	20	0.084	21	10	50
19	21	0.082	21	20	0

**Table 2:** Parametric information regarding branches and constant power loads in the bipolar DC 33-bus network (all powers in kW).

Node *j*	Node *k*	$R_{\mathrm{jk}}$ (Ω)	$P_{d,k}^{p}$	$P_{d,k}^{n}$	$P_{d,k}^{p-n}$
1	2	0.0922	100	150	0
2	3	0.4930	90	75	0
3	4	0.3660	120	100	0
4	5	0.3811	60	90	0
5	6	0.8190	60	0	200
6	7	0.1872	100	50	150
7	8	1.7114	100	0	0
8	9	1.0300	60	70	100
9	10	1.0400	60	80	25
10	11	0.1966	45	0	0
11	12	0.3744	60	90	0
12	13	1.4680	60	60	100
13	14	0.5416	120	100	200
14	15	0.5910	60	30	50
15	16	0.7463	110	0	350
16	17	1.2890	60	90	0
17	18	0.7320	90	45	0
2	19	0.1640	90	150	0
19	20	1.5042	150	50	115
20	21	0.4095	0	90	0
21	22	0.7089	0	90	145
3	23	0.4512	90	110	35
23	24	0.8980	120	0	40
24	25	0.8960	150	100	100
6	26	0.2030	60	80	0
26	27	0.2842	60	0	225
27	28	1.0590	0	0	130
28	29	0.8042	120	75	65
29	30	0.5075	100	100	0
30	31	0.9744	50	150	125
31	32	0.3105	175	100	75
32	33	0.3410	95	60	120

**Table 3:** Parametric information regarding branches and constant power loads in the bipolar DC 85-bus network (all powers in kW).

Node *j*	Node *k*	$R_{\mathrm{jk}}$ (Ω)	$P_{\mathrm{dk}}^{p}$	$P_{\mathrm{dk}}^{p}$	$P_{\mathrm{dk}}^{p-n}$	Node *j*	Node *k*	$R_{\mathrm{jk}}$ (Ω)	$P_{\mathrm{dk}}^{p}$	$P_{\mathrm{dk}}^{p}$	$P_{\mathrm{dk}}^{p-n}$
1	2	0.108	0	0	10.075	34	44	1.002	17.64	17.995	0
2	3	0.163	50	0	40.35	44	45	0.911	50	17.64	17.995
3	4	0.217	28	28.565	0	45	46	0.911	25	17.64	17.995
4	5	0.108	100	50	0	46	47	0.546	7	7.14	10
5	6	0.435	17.64	17.995	25.18	35	48	0.637	0	10	0
6	7	0.272	0	8.625	0	48	49	0.182	0	0	25
7	8	1.197	17.64	17.995	30.29	49	50	0.364	18.14	0	18.505
8	9	0.108	17.8	350	40.46	50	51	0.455	28	28.565	0
9	10	0.598	0	100	0	48	52	1.366	30	0	15
10	11	0.544	28	28.565	0	52	53	0.455	17.64	35	17.995
11	12	0.544	0	40	45	53	54	0.546	28	30	28.565
12	13	0.598	45	40	22.5	52	55	0.546	38	0	48.565
13	14	0.272	17.64	17.995	35.13	49	56	0.546	7	40	32.14
14	15	0.326	17.64	17.995	20.175	9	57	0.273	48	35.065	10
2	16	0.728	17.64	67.5	33.49	57	58	0.819	0	50	0
3	17	0.455	56.1	57.15	50.25	58	59	0.182	18	28.565	25
5	18	0.820	28	28.565	200	58	60	0.546	28	43.565	0
18	19	0.637	28	28.565	10	60	61	0.728	18	28.565	30
19	20	0.455	17.64	17.995	150	61	62	1.002	12.5	29.065	0
20	21	0.819	17.64	70	152.5	60	63	0.182	7	7.14	5
21	22	1.548	17.64	17.995	30	63	64	0.728	0	0	50
19	23	0.182	28	75	28.565	64	65	0.182	12.5	25	37.5
7	24	0.910	0	17.64	17.995	65	66	0.182	40	48.565	33
8	25	0.455	17.64	17.995	50	64	67	0.455	0	0	0
25	26	0.364	0	28	28.565	67	68	0.910	0	0	0
26	27	0.546	110	75	175	68	69	1.092	13	18.565	25
27	28	0.273	28	125	28.565	69	70	0.455	0	20	0
28	29	0.546	0	50	75	70	71	0.546	17.64	38.275	17.995
29	30	0.546	17.64	0	17.995	67	72	0.182	28	13.565	0
30	31	0.273	17.64	17.995	0	68	73	1.184	30	0	0
31	32	0.182	0	175	0	73	74	0.273	28	50	28.565
32	33	0.182	7	7.14	12.5	73	75	1.002	17.64	6.23	17.995
33	34	0.819	0	0	0	70	76	0.546	38	48.565	0
34	35	0.637	0	0	50	65	77	0.091	7	17.14	25
35	36	0.182	17.64	0	17.995	10	78	0.637	28	6	28.565
26	37	0.364	28	30	28.565	67	79	0.546	17.64	42.995	0
27	38	1.002	28	28.565	25	12	80	0.728	28	28.565	30
29	39	0.546	0	28	28.565	80	81	0.364	45	0	75
32	40	0.455	17.64	0	17.995	81	82	0.091	28	53.75	0
40	41	1.002	10	0	0	81	83	1.092	12.64	32.995	62.5
41	42	0.273	17.64	25	17.995	83	84	1.002	62	72.2	0
41	43	0.455	17.64	17.995	0	13	85	0.819	10	10	10

**Table 4:** Comparative analysis between Broyden’s method and literature reports.

	Neutral Wire Floating			Neutral Wire Solidly Grounded		
Bipolar DC 21-Bus Network						
Method	Losses (kW)	Iterations	Time (ms)	Losses (kW)	Iterations	Time (ms)
SAPF	95.4237	13	0.5275	91.2701	10	0.4911
TBPF	95.4237	13	0.8340	91.2701	10	0.7672
HAPF	95.4237	13	1.5542	91.2701	4	1.0212
Proposed	95.4237	7	1.1593	91.2701	11	0.8776
Bipolar DC 33-bus network						
SAPF	344.4797	11	1.2537	334.4168	9	1.0594
TBPF	344.4797	11	2.1875	334.4168	9	2.0171
HAPF	344.4797	11	5.5386	334.4168	4	3.4675
Proposed	344.4797	7	2.5657	334.4168	11	1.9950
Bipolar DC 85-bus network						
SAPF	489.5759	13	6.4419	452.2981	10	6.3261
SAPF	489.5759	13	8.4913	452.2981	10	8.3822
HAPF	489.5759	13	15.1654	452.2981	4	10.5698
Proposed	489.5759	7	12.4023	452.2981	11	8.7853

## Data Availability

No new data were created or analyzed in this study. Data sharing does not apply to this article.

## Nomenclature

Indices	
p,o,n	Superscripts associated with poles
0	Superscripts associated with the initial value
*t*	Superscripts associated with the iteration counter
j,k	Subscripts associated with nodes
*g*	Subscripts associated with the generators
*d*	Subscripts associated with the demands
Parameters	
${G_{jk}}^{pr}$	Value of the conductance matrix that associates nodes *j* and *k* between poles *p* and *r* (S)
$G_{gg}$	Value of the conductance matrix associated only with generators (S)
$G_{gd}$, $G_{dg}$	Value of the conductance matrix associated with generators and demands (S)
$G_{dd}$	Value of the conductance matrix associated only with demands (S)
$P_{d,k}^{p-n}$	Bipolar constant power consumption connected between the positive and negative poles and node *k* (W)
$P_{d,k}^{p}$	Monopolar constant power consumption at node *k* for the positive pole *p* (W)
$P_{d,k}^{n}$	Monopolar constant power consumption at node *k* for the negative pole *n* (W)
$V_{\mathrm{nom}}$	Nominal voltage at the substation terminal (V)
$\nabla f\left(x\right)$	Gradient of the function f(x)
Sets	
P	Set that contains all the poles in the network, i.e., $\left\{p,o,n\right\}$
N	Set that contains all nodes in the network
Variables	
$V_{k}^{r}$	Voltage value at node *k* for the $r^{th}$ pole (V)
$I_{g,k}^{p}$	Current injection in the slack source at node *k* for the positive pole *p* (A)
$I_{g,k}^{o}$	Current injection in the slack source at node *k* for the neutral pole *o* (A)
$I_{g,k}^{n}$	Current injection in the slack source at node *k* for the negative pole *n* (A)
$I_{d,k}^{p}$	Current consumption at node *k* for the positive pole *p* (A)
$I_{d,k}^{o}$	Current consumption at node *k* for the neutral pole *p* (A)
$I_{d,k}^{n}$	Current consumption at node *k* for the negative pole *p* (A)
$I_{d,k}^{\mathrm{ground}}$	Current consumption at node *k* for the ground (A)
$v_{g}$	Vector that contains the voltage in the slack source (V)
$I_{g}$	Vector that contains the current injection in the slack source (A)
$V_{d}$	Vector that contains the voltage in the demands (V)
$I_{d}$	Vector that contains the current consumptions (A)
$I_{d}^{B}$	Vector that contains the current in bipolar loads (A)
$I_{d}^{M}$	Vector that contains the current in monopolar loads (A)
$A^{t}$	Jacobian matrix in iteration *t*
